# The nuclear envelope and nuclear pore complexes in neurodegenerative diseases

**DOI:** 10.3389/fcell.2025.1550859

**Published:** 2025-05-13

**Authors:** Tingyan Wu, Haochen Xu, Lei Cheng, Ruoxin Wu, Fuzheng Guo, Xi Chen

**Affiliations:** ^1^ Institute of Neurology, Sichuan Provincial People’s Hospital, School of Medicine, University of Electronic Science and Technology of China, Chengdu, China; ^2^ Chinese Academy of Sciences Sichuan Translational Medicine Research Hospital, University of Electronic Science and Technology of China, Chengdu, China; ^3^ Department of Neurology, School of Medicine, University of California, Sacramento, CA, United States

**Keywords:** nuclear envelope, nuclear pore complexes, nucleoporins, neurodegenerative diseases, nuclear transport

## Abstract

The nuclear envelope (NE) and nuclear pore complexes (NPCs) play a critical role in maintaining the balance between the nucleus and cytoplasm, which is essential for the structural integrity and gene regulatory functions of eukaryotic cells. Disruptions in the nucleocytoplasmic trafficking mediated by the NE and NPCs can compromise nuclear integrity and transport homeostasis, ultimately threatening cellular viability. Recent research has highlighted a strong link between dysfunction of the NE and NPCs and the onset of neurodegenerative disorders. In this review, we summarize the current understanding of how impairments in nuclear transport contribute to the pathogenesis of neurodegenerative diseases, with a particular focus on the NE and NPCs. We aim to shed light on the intricate relationship between these molecular gatekeepers and the pathological cascade leading to neuronal degeneration, while also exploring potential strategies to restore cellular homeostasis and mitigate the progression of these devastating neurological conditions.

## Introduction

In eukaryotic cells, the nucleus is compartmentalized by the nuclear envelope (NE), a dynamic structure that regulates communication between the nucleus and cytoplasm. This selective barrier maintains genome integrity while enabling the exchange of macromolecules essential for cellular function (Rush et al., 2023). Central to this process is the nuclear pore complex (NPC), a massive protein assembly embedded within the NE that governs nucleocytoplasmic transport (NCT). By facilitating the selective and energy-dependent passage of RNAs, proteins, and signaling molecules, the NPC ensures proper gene expression, signal transduction, and cellular homeostasis (Yang et al., 2023). Beyond its role in transport, the NPC also contributes to genome organization, transcriptional regulation, and stress responses, linking nuclear function to broader cellular processes (Liu and Hetzer, 2022).

### Nuclear envelope

**FIGURE 1:**
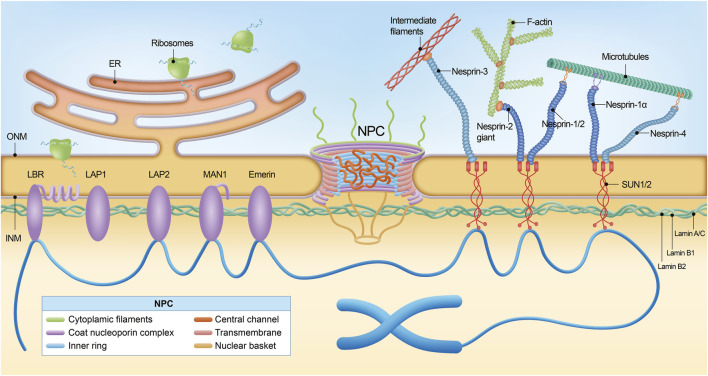
The nuclear pore complex and nuclear envelope. The nuclear pore complex (NPC) is a large macromolecular structure and is comprised of six subdomains: the cytoplasmic filaments, the coat nucleoporin complex, the inner ring, the central channel, the transmembrane nucleoporins, and the nuclear basket. Many phenylalanine-glycine (FG)-Nups (indicated by an asterisk) are found in the central channel. The NPC sits within the double membrane of the nucleus, and the NPC core demonstrates two-fold symmetry across the nuclear envelope, with the asymmetric cytoplasmic filaments and nuclear basket Nups projecting into their corresponding cellular compartments. Nuclear lamina is localized between the inner nuclear membrane and chromatin. Schematic representation of lamin interaction with inner nuclear membrane proteins, the most important of which are MAN1, LAP2, SUN1/2, Emerin, and LBR. The NPC spans both the inner nuclear membrane and the outer nuclear membrane. Via SUN1/2 and the nesprins interacting with them, located in the outer nuclear membrane, lamins cooperate with cytoskeleton components, namely, filamentous actin (F-actin), microtubules (MTs), and intermediate filaments (IFs).

NE is a continuous membrane system composed of two distinct phospholipid bilayers: the outer nuclear membrane (ONM) and the inner nuclear membrane (INM) (Figure 1) (Stewart et al., 2007). The ONM is contiguous with the endoplasmic reticulum and studded with ribosomes on the cytoplasmic side. Embedded within the ONM are various transmembrane proteins that facilitate cellular processes such as migration, division, and differentiation. Among these, nesprins, characterized by the Klarsicht/ANC-1/Syne-1 homology (KASH) domain, are integral components of the LINC (Linker of Nucleoskeleton and Cytoskeleton) complex, which also includes SUN (Sad1 and UNC-84) domain-containing proteins, SUN1 and SUN2 (Manda et al., 2023; Turgay et al., 2010). These LINC complexes establish direct connections between cytoskeletal structures (including microtubules, intermediate filaments, and actin) and the lamina, providing efficient transmission between the cytoskeleton and nucleus and supporting cellular integrity and function (Méjat and Misteli, 2010; Smith et al., 2022). In specific, LINC complexes have been reported to interact with cytoplasmic stress fibers, composed of bundled F-actin filaments anchored at focal adhesions. This interaction forms a nuclear actin “cap,” a specialized structure that regulates nuclear shape and facilitates the reorganization of lamins (Davidson and Cadot, 2021; Sobo et al., 2024). In addition, reduced levels of LINC complex have been reported in Amyotrophic lateral sclerosis (ALS) (Sirtori et al., 2024).

The INM serves as a protective barrier for the nucleus in eukaryotic cells and interacts with chromatin and the nuclear lamina. INM houses a curated set of proteins integral to various nuclear functions, including Emerin, MAN1, the Lamin B receptor (LBR), and Lamina-associated polypeptide 1/2 (LAP1/2) (Figure 1) (Barton et al., 2015; Sobo et al., 2024). Emerin, encoded by the *EMD* gene, interacts with multiple nuclear envelope proteins, most notably lamin A/C, and also binds to chromatin (Berk et al., 2014; Liddane and Holaska, 2021). These interactions are critical for preserving nuclear architecture and stability, facilitating gene regulation, and modulating cell signaling pathways. Mutations in the *EMD* gene can lead to either the loss or dysfunction of Emerin, with particularly notable implications for the development of Emery-Dreifuss muscular dystrophy (EDMD), a disorder marked by defects in cardiac and skeletal muscle function (Bengtsson and Wilson, 2004; Morris and Manilal, 1999). MAN1 belongs to the LEM domain family (Borah et al., 2022), and LBR interacts with lamin B, critical for maintaining the mechanical stability of the nucleus and chromatin organization (Nikolakaki et al., 2017).

### Nuclear pore complex and nucleoporins

**FIGURE 2:**
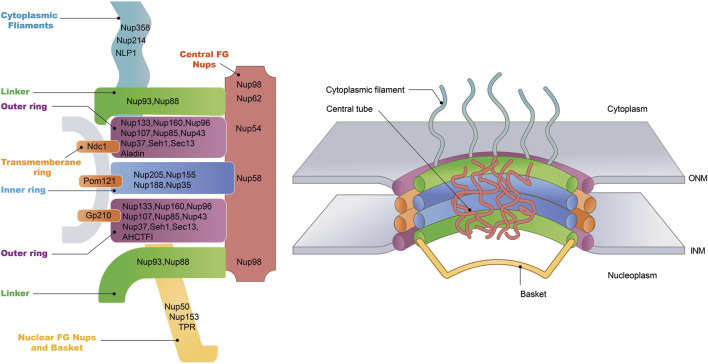
Nuclear pore complex structure. The NPC is a cylindrical structure composed of eight radial spokes surrounding a central channel, which serves as a passageway between the nucleoplasm and the cytoplasm. The ONM and the INM of the nuclear envelope converge to form grommet-like openings where the NPC is embedded. The NPC is anchored to the nuclear envelope by a transmembrane ring structure, which connects to the core scaffold comprising both inner and outer ring components. Linker Nups are crucial for maintaining NPC stability by anchoring phenylalanine-glycine (FG) Nups, facilitating selective transport. The NPC is associated with peripheral structures, including cytoplasmic filaments and nuclear basket. The specific nucleoporins that constitute each NPC substructure have been listed.

ONM and INM, the two concentric membranes converge at the sites where NPCs are inserted. NPCs are large protein structures mediating macromolecular trafficking between the nuclear and cytoplasmic compartments (Figures 1, 2) (Raices and D’Angelo, 2012). Electron microscopy studies have revealed that NPCs possess a doughnut-shaped structure, comprising eight spokes with a molecular weight ranging from roughly 60 to 125 MD; the mass is smaller in yeast at approximately 60 MD, whereas in humans, it ranges from 90 to 125 MD (Ryan and Wente, 2000). Measurements of the NPCs reveal a minimal pore diameter that ranges from approximately 40–50 nm and extend longitudinally to a length varying between 40 and 95 nm (Musser and Grünwald, 2016). Typically, the nucleus of a vertebrate cell contains about 2,000–3,000 NPCs, underscoring the centrality of these structures in facilitating the dynamic flow of cellular content (Knockenhauer and Schwartz, 2016).

NPC is an elaborate complex composed of approximately 30 distinct protein subunits, collectively termed nucleoporins (Nups) (Figure 2) (Jühlen and Fahrenkrog, 2023). These Nups are highly conserved across eukaryotic species, reflecting the essential role of the NPC in cellular function. Nups are typically categorized into two principal groups distinguished by their roles and positions within the NPCs. The first group encompasses the core scaffold components of the NPC, which are stably integrated within the NE. Among these, the Nup107–Nup160 complex, also known as Y-complex, constitutes a significant portion of the outer ring structure of the NPCs and represents the most comprehensively studied subcomplex within this category (Lin and Hoelz, 2019). Of particular interest is Nup133, an integral constituent of the Nup107-160 complex, which has been demonstrated to be indispensable for neuronal differentiation. Evidence from studies on mouse embryonic stem cells (ESCs) with Nup133 depletion shows significant impairment in their capacity to differentiate into neural lineages, alongside the retention of atypical pluripotency features (Lupu et al., 2008). Additionally, Nup35, Nup188/Nup205 and Nup155 are the principal components that constitute the inner ring (Li et al., 2022). In contrast to the defined Y-shaped architecture of the outer ring, inner ring subcomplexes observed by negative-stain EM are highly heterogeneous in conformation (Kosinski et al., 2016).

The second group of Nups comprises the mobile Nups, which are characterized by the presence of a disordered phenylalanine-glycine (FG) repeat domain (Davis et al., 2022; Denning et al., 2003). These FG-repeat Nups, including Nup62, Nup98, and Nup54, are essential components of the NPC, where they occupy the central channel spanning the nuclear envelope. The FG-repeat domain consists of hydrophobic phenylalanine (F) and glycine (G) residues separated by short linker sequences. This flexible, disordered structure enables FG-Nups to form a dynamic, gel-like meshwork within the NPC, which is critical for its selective permeability.

The FG-repeat Nups play a pivotal role in mediating the transport of molecules across the nuclear envelope (Chen et al., 2025). They interact with soluble transport receptors, such as importins and exportins, which facilitate the movement of cargo proteins between the cytoplasm and the nucleus. The selective permeability of the NPC is regulated by these interactions, as the FG-repeat domains create a barrier that restricts the uncontrolled diffusion of large macromolecules while allowing the efficient transport of cargo through the central channel (Fare and Rothstein, 2024). Furthermore, FG-Nups contribute to the structural integrity of the NPC, which is essential for proper cellular function. Disruptions in FG-repeat Nup function can impair NCT, leading to cellular dysfunction and disease.

### Neuronal nucleoporins

In the post-mitotic cells, the complete disassembly of NPCs poses a substantial risk to nuclear integrity, potentially leading to partial NE breakdown or a temporary disruption in the segregation between nuclear and cytoplasmic contents. Unlike other large protein assemblies, such as ribosomes or proteasomes, NPCs exhibit a unique turnover rate (D’Angelo et al., 2009; Toyama et al., 2013). A notable finding has shown that approximately 25% of Nup205 complexes remain unchanged for up to a year in non-dividing cells, highlighting a remarkably slow turnover rate there (Toyama et al., 2013). Further analysis of cultured postmitotic muscle cells revealed that POM121 exhibits a rapid exchange within 2–3 days. On the other hand, no detectable exchange of Nup93, a component of the Nup205 complex, was observed over a 2-week duration (Toyama et al., 2019). Additionally, two components of the Nup107 complex exhibited slightly faster exchange rates, albeit with some variation (Toyama et al., 2019).

The diverse turnover rates of Nups in post-mitotic cells suggest a finely tuned balance between structural stability and functional adaptability, with critical implications for nuclear integrity, cellular aging, and differentiation. Unlike ribosomes or proteasomes, which undergo coordinated turnover, NPCs exhibit a mosaic of exchange rates—some Nups, like Nup205 and Nup93, persist unchanged for weeks or even years, forming a stable scaffold that safeguards the nuclear envelope against catastrophic disassembly. This remarkable longevity may be essential for maintaining the selective barrier between nucleus and cytoplasm in long-lived cells, but it also renders these Nups vulnerable to cumulative damage, potentially contributing to age-related decline in nuclear transport efficiency (Hakhverdyan et al., 2021).

Conversely, the rapid turnover of Nups like POM121 hints at a dynamic layer of regulation, allowing cells to adjust nuclear permeability or repair localized damage without dismantling the entire pore complex. Such differential turnover could be particularly important in post-mitotic cells, which cannot rely on mitotic NPC reassembly to refresh their nuclear pores. Instead, they may employ a piecemeal maintenance strategy—preserving core structural elements while selectively replacing peripheral or stress-exposed components. This could explain why certain Nups within the same complex (e.g., Nup107) exhibit different exchange rates, enabling functional plasticity without compromising overall architecture.

The biological significance of these dynamics may extend beyond mere maintenance. In differentiating cells, stable Nup populations could help lock in cell-type-specific transport properties, ensuring consistent gene expression profiles, while more dynamic Nups might allow adaptive responses to metabolic or environmental changes. Meanwhile, the extreme longevity of some Nups raises intriguing questions about how they evade degradation pathways—whether through protective modifications, privileged localization, or resistance to proteostasis machinery. Understanding these mechanisms could shed light on broader proteostatic challenges in aging, particularly in neurons or muscle cells, where NPC dysfunction has been linked to degenerative diseases.

Ultimately, the heterogeneous turnover of Nups suggests that NPCs are not static structures but rather mosaics of stability and dynamism, optimized for the unique demands of post-mitotic life. Future work dissecting the regulatory logic behind these turnover rates—and their failure in aging—could reveal new strategies to enhance cellular resilience or counteract age-related nuclear defects.

### RNA transport through the nuclear pore

**FIGURE 3:**
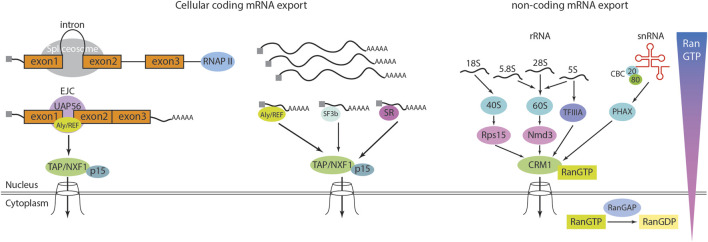
RNA export from nucleus. Cellular mRNA export is mediated by the heterodimeric export receptor TAP/NXF1-p15. Unlike karyopherins, TAP/NXF1 is structurally distinct and does not require Ran-GTP for its function. The export of mRNA is closely linked to earlier stages of RNA biogenesis. In metazoans, most mRNAs contain introns, and splicing occurs co-transcriptionally while the pre-mRNA is being synthesized by RNA polymerase II (RNAP II). Processing factors associate with the C-terminal domain of RNAP II and travel along with the elongating transcription complex to ensure proper RNA maturation. During splicing, the splicing protein UAP56 recruits the export adapter Aly/REF to a position just upstream of the exon 1–exon 2 junction. Aly/REF is a key component of the exon junction complex (EJC). The mature mRNA, which is capped at the 5′end, polyadenylated at the 3′end, and decorated with EJCs at exon-exon junctions, is subsequently exported *via* TAP/NXF1-p15, which interacts with the NPC. While most cellular mRNAs rely on this pathway, intronless mRNAs can also be exported through Aly/REF or alternative export adapters such as SR and SF3b proteins, suggesting the existence of additional, yet unidentified, export mechanisms. The karyopherin/exportin family mediates the export of non-coding RNAs. In general, this process requires karyopherin/exportin to bind Ran-GTP, allowing it to associate with its cargo. Upon reaching the cytoplasm, RanGAP converts Ran-GTP to Ran-GDP, leading to cargo release. For small nuclear RNAs (snRNAs), the cap-binding complex (CBC), a heterodimer composed of 80 kDa and 20 kDa subunits, binds to the 5′cap. CBC then associates with the phosphorylated adapter of RNA export (PHAX), which in turn recruits CRM1, a member of the karyopherin family, to facilitate export. Ribosomal RNAs (rRNAs) transcribed by RNA polymerase I undergo processing to generate 18S, 5.8S, and 28S rRNAs. The 18S rRNA assembles with ribosomal proteins to form the 40S pre-ribosomal complex, which is exported *via* CRM1-Ran-GTP through its interaction with Rps15a. Meanwhile, the 5.8S, 28S, and 5S rRNAs form the 60S pre-ribosomal complex, which is exported *via* CRM1-Ran-GTP through Nmd3. Additionally, a subset of 5S rRNA binds to TFIIIA, which acts as a bridge to CRM1, facilitating its export.

In eukaryotes, the export of RNA from the nucleus is a vital stage of gene expression, ensuring the delivery of genetic information to the cytoplasm for protein synthesis (Figure 3). RNA forms a complex with multiple proteins, resulting in the formation of a messenger ribonucleoprotein (mRNP) complex (Soheilypour and Mofrad, 2018). Before exiting the nucleus, this complex undergoes rigorous quality control by export receptors, such as CRM1 (Chromosome Region Maintenance 1) or TAP/NXF1 (Nuclear Export Factor 1) (Ding and Sepehrimanesh, 2021; Ossareh-Nazari et al., 1997). Concurrently, FG-repeat Nups act as a selective barrier to facilitate the translocation process by allowing the mRNA-export receptor complex to pass through, while restricting the uncontrolled diffusion of larger molecules. TAP/NXF1-dependent mRNA export plays a critical role in the export of spliced mRNA and relies significantly on Aly/REF, SF3b, and SR to facilitate the final translocation through the NPC. In contrast, Crm1-dependent export is particularly essential for the export of snRNA (small nuclear RNA) and rRNA (ribosome RNA). Unlike TAP/NXF1-mediated export, this pathway requires direct interaction with the RanGTP gradient (Mougel et al., 2020). RanGTP, a small GTPase that transitions between its GTP-bound and GDP-bound conformations, is fundamental for establishing and maintaining the directionality of nuclear transport, thereby facilitating the efficient export of mRNA from the nucleus to the cytoplasm (Bonnet and Palancade, 2014).

TPR, located in the nuclear basket of the NPC, serves as the primary docking site for mRNPs and plays a pivotal role in the final stages of mRNA export. It facilitates this process by interacting with export receptors, such as NXF1/TAP, and RNA helicases like Dbp5, which promote the translocation of mRNPs through the nuclear pore. Additionally, Nup153 is essential for initiating mRNA export through the NPC’s central channel. It anchors mRNPs to the NPC and recruits critical export factors, such as TAP, during the early stages of export (Li et al., 2021; Singh et al., 2024). Nup50, another component of the nuclear basket, has been implicated in NCT but appears to play a less critical role in the direct docking or export of mRNPs compared to TPR and Nup153. While Nup50 is essential for the disassembly of importin-cargo complexes and the recycling of transport receptors, its contribution to mRNA export is minimal. However, Nup50 has been linked to various pathologies, particularly those involving defects in NCT, suggesting a broader, albeit less understood, role in cellular processes. For instance, Nup50 could contribute to mRNP assembly, stability, or the nuclear import of proteins required for mRNA export. Alternatively, its dysfunction may disrupt broader NPC activities, such as gene expression regulation or protein homeostasis, leading to pathological consequences. This discrepancy between its limited role in mRNA export and its significant contribution to disease highlights the need for further investigation into how Nup50 may influence cellular pathways beyond its established functions. Together, this elaborate regulatory system ensures the fidelity and efficiency of mRNA export, which is critical for proper cellular function and homeostasis. These interactions, along with the multiple quality control steps that mRNPs undergo on different components of the NPC, collectively ensure that only the correct mRNA is exported to the cytoplasm.

### Protein transport through the nuclear pore

The nuclear localization signal (NLS) and the nuclear export signal (NES) are pivotal sequences that govern the bidirectional trafficking of cargo proteins between the nucleus and cytoplasm (Wing et al., 2022). The canonical nuclear import pathway commences with the binding of an NLS-bearing cargo protein to importins, forming an importin-cargo complex. This complex is subsequently chaperoned with importin β, facilitated by interactions with FG-repeat Nups through the NPCs (Baade and Kehlenbach, 2019). Upon reaching the nucleus, the import process concludes with the RanGTP-mediated release of the cargo protein. Specifically, RanGTP binds to importin β on the nuclear side of the NPCs, triggering the dissociation and subsequent release of the cargo into the nucleus. The replenishment of RanGTP within the nucleus is achieved through the import of RanGDP. This import is facilitated by nuclear transport factor 2 (NTF2), which specifically binds RanGDP and escorts it into the nucleus *via* the NPCs (Bischoff and Ponstingl, 1991; Smith et al., 1998). Additionally, by promoting the conversion of RanGDP to RanGTP, NTF2 sustains the Ran gradient across the nuclear envelope, which is a prerequisite for the directionality of nuclear transport.

The export of proteins from the nucleus to the cytosol is orchestrated by a distinct group of proteins known as exportins. These specialized receptors selectively engage with the NES embedded within the cargo protein, culminating in the formation of a stable exportin-cargo complex (Bitetto and Di Fonzo, 2020). Subsequently, this complex is transported through the NPCs to reach the cytosol. Upon arrival in the cytosol, RanGTP binds to the exportin, triggering a conformational shift, which in turn catalyzes the detachment of the cargo protein. The liberated cargo is now free to participate in cytosolic activities. The Ran GTPase-activating protein (RanGAP) converts any RanGTP entering the cytoplasm to RanGDP by stimulating Ran to hydrolyze its bound GTP. The regulated transport of proteins across the nuclear envelope is essential for numerous cellular processes, including gene regulation, signal transduction, and cell cycle coordination. Disruptions or abnormalities in this highly regulated transport system can have profound consequences, potentially leading to a range of diseases and impairing normal cellular functions (Pasha et al., 2021).

### Novel assays that illuminate nuclear envelope and nuclear pore complex dynamics

Several novel assays have been developed to study the NPC and NE, particularly in the context of their structure, function, and dynamics. Here are some cutting-edge approaches.

Our understanding of human NPC architecture has largely been shaped by cryo-electron tomography (cryo-ET) studies of NPCs isolated from purified NE, achieving resolutions of 2–6 nm (Bui et al., 2013). However, the advances in cryo-focused-ion-beam (cryo-FIB) milling now enable *in situ* structural analysis of macromolecular complexes like NPCs within their native cellular environments (Villa et al., 2013). Comparative studies highlight significant quantitative differences between NPCs observed in cryo-FIB-milled yeast, algae, and human cells *versus* earlier models derived from purified nuclear envelopes (Allegretti et al., 2020; Mosalaganti et al., 2018; Zila et al., 2021). Notably, in cryo-FIB-milled human DLD-1 cells, the inner ring is 16 nm wider than previously reported, increasing the central channel volume by 75% and driving the reorganization of cytoplasmic and nucleoplasmic rings (Schuller et al., 2021). These structural deviations are further accompanied by asymmetric membrane curvature around the inner ring complex, challenging the long-standing assumption of NPC symmetry. Such findings underscore the critical importance of *in situ* quantification, as purification methods may introduce artifacts that distort NPC dimensions. Future high-resolution cryo-ET workflows, integrated with molecular dynamics simulations, could elucidate how these nanometer-scale structural variations influence transport kinetics and selectivity, bridging quantitative structural biology with functional insights.

Single-molecule tracking (SMT) enables real-time visualization of individual molecules transiting the NPC, revealing dynamic transport mechanisms. Early breakthroughs, such as Narrow-Field Epifluorescence Microscopy (Yang et al., 2004), quantified passive and facilitated transport kinetics in permeabilized cells, measuring cargo dwell times (∼5–50 ms) and diffusion rates. Subsequent techniques (Dange et al., 2008; Lowe et al., 2010) advanced spatial-temporal resolution but were limited to 1D analysis, missing 3D transport dynamics. To address this, single-point edge-excitation sub-diffraction (SPEED) microscopy (Ma et al., 2013; Ma and Yang, 2010) was developed, achieving <10 nm 3D localization precision at speeds up to 5,000 frames per second. This revealed quantitative transport disparities: pre-ribosomal particles exhibit ∼66% nuclear export efficiency (vs. lower rates for mRNAs), with CRM1 inhibition reducing pre-60S/pre-40S export efficiency by 11–17-fold (Junod et al., 2023). Cooperative CRM1 binding accelerates export, shortening transit times by ∼30% for pre-ribosomal subunits and other cargos. These studies highlight how nanometer-scale spatial resolution and millisecond temporal precision resolve mechanistic principles of NPC transport.

Optogenetic and photoactivatable systems have emerged as powerful tools to dissect NPC dynamics with spatiotemporal precision. Niopek et al. (2016) developed LEXY, a light-inducible nuclear export system that offers precise, quantitative control over the nucleocytoplasmic distribution of proteins (Niopek et al., 2016). LEXY is based on an engineered LOV2 domain from Avena sativa phototropin-1, in which the C-terminal Jα helix is modified to include a photocaged nuclear export signal (NES). In the dark, the NES remains hidden, keeping the tagged protein nuclear; however, blue light triggers a conformational change that rapidly exposes the NES, resulting in fast (half-times below 1 min) and fully reversible nuclear export. The system’s robustness is further highlighted by a chromatin-anchored variant that sequesters endogenous CRM1 receptors, effectively inhibiting nuclear export in a light-dependent manner. This innovative optogenetic tool not only allows researchers to dissect the dynamics of NCT with high spatial and temporal resolution but also provides a versatile platform for controlling the activity of key regulatory proteins (e.g., p53), with promising applications in synthetic and cell biology.

### Unresolved questions regarding the nuclear envelope and nuclear pore complex

Despite significant progress in elucidating the structure and fundamental functions of the NE and NPCs, several key questions remain unresolved. One major challenge lies in understanding the orchestration of NPC assembly and disassembly, particularly how cells integrate signals to insert new NPCs into an intact nuclear envelope and how quality control systems identify, repair, or remove defective complexes. While much is known about the selective transport mechanism mediated by FG-repeat nucleoporins, it remains unclear how their dynamic phase behavior precisely facilitates rapid and regulated cargo exchange. Additionally, the long-term maintenance of NPC integrity in post-mitotic cells, which must sustain thousands of these massive structures over extended periods, raises questions about the mechanisms that detect subtle functional declines and initiate remodeling or turnover. Finally, the interplay between NPC composition and broader cellular processes, such as gene regulation and stress responses, remains an active area of investigation. Addressing these unknowns will not only advance our fundamental understanding of NCT but also shed light on how NPC dysfunction contributes to aging and disease.

Emerging evidence suggests that impaired NCT, nuclear envelope instability, and mislocalization of essential proteins are common features in several neurodegenerative disorders, ALS (Cook et al., 2020; Gleixner et al., 2022), Alzheimer’s disease (AD) (Lee et al., 2006; Sheffield et al., 2006), Huntington’s disease (HD) (Hosp et al., 2015; Suhr et al., 2001), and Parkinson’s disease (PD) (Um et al., 2006; Xia et al., 2008). These disruptions not only compromise neuronal function but also exacerbate disease progression by impairing stress responses and accelerating cellular degeneration. In the following sections, we will examine how NE and NPC dysfunction contribute to the molecular pathology of these disorders, highlighting key mechanisms and their potential as therapeutic targets.”

## Nuclear pore complex in neurodegenerative diseases

### Nuclear pore complex and ALS

ALS, a relentlessly progressive neurodegenerative disorder, is characterized by the selective degeneration of upper and lower motor neurons in the cortex, brainstem, and spinal cord, culminating in muscle atrophy, paralysis, and fatal respiratory failure within 3–5 years of onset (Masrori and Van Damme, 2020; Vahsen et al., 2021). While 5%–10% of cases are familial (fALS), driven by mutations in genes such as C9orf72, TDP-43, SOD1 and FUS, the majority are sporadic (sALS) with elusive triggers. A unifying pathological hallmark across ALS subtypes is the dysregulation of NCT, which is orchestrated by NPCs. Emerging evidence implicates NPC dysfunction as a central mechanism underlying ALS pathogenesis, with aberrant Nup localization, aggregation, and depletion disrupting the nuclear-cytoplasmic trafficking of critical proteins and RNAs (Cristi et al., 2023). This breakdown in NCT not only compromises motor neuron survival but also illuminates shared pathways between genetic and sporadic forms of the disease.

The G_4_C_2_ hexanucleotide repeat expansion (HRE) in C9orf72, the most common genetic cause of ALS, drives pathogenesis through a toxic gain-of-function mechanism involving both dipeptide repeat proteins (DPRs) and HRE RNA toxicity, ultimately converging on NPC dysfunction. DPRs such as poly-GR and poly-PR, produced *via* repeat-associated non-AUG (RAN) translation, disrupt the RanGTP gradient required for NCT, thereby impairing nuclear import and exacerbating the cytoplasmic mislocalization of TDP-43—an established pathological hallmark of ALS (Allegretti et al., 2020; Khosravi et al., 2017). In parallel, HRE RNA directly interacts with RanGAP, altering its localization and leading to nuclear import deficits in both fly models and iPSC-derived neurons (Zhang et al., 2015). Further mechanistic investigations reveal that RanGAP mislocalization in C9orf72-ALS may stem from the depletion of POM121, a transmembrane nucleoporin essential for NPC assembly, as observed in iPSC-derived motor neurons from ALS patients (Coyne et al., 2020). Indeed, a broad reduction in nucleoporin expression has been consistently reported in ALS models, although the precise underlying mechanism remains unclear. Specifically, iPSC-derived motor neurons harboring the C9orf72 mutation exhibit a decline in multiple nucleoporins, including TPR, Nup98, NDC1, Nup107, Nup133, Nup50, GP210, and POM121 (Coyne et al., 2020). Notably, with the exception of GP210, this same set of nucleoporins is also depleted in *postmortem* motor cortex tissues from ALS patients, reinforcing the pathological relevance of NPC dysfunction in ALS and underscoring the utility of endogenous model systems for mechanistic studies (Coyne et al., 2020). One proposed mechanism underlying nucleoporin loss involves the aberrant nuclear accumulation of charged multivesicular body protein 7 (CHMP7), a key component of the Endosomal Sorting Complex Required for Transport III (ESCRT-III) (Coyne et al., 2021) *via* CHMP2B (Keeley et al., 2024). Further mechanistic studies reveal that SmD1, a component of the survival of motor neuron (SMN) complex, influences the subcellular localization of CHMP7. SmD1 is a small nuclear ribonucleoprotein (snRNP) involved in the processing of small nuclear RNAs (snRNAs) and the regulation of splicing factor mRNAs in motor neurons. Notably, overexpression of SmD1 in ALS patient-derived iPSC motor neurons restored the cytoplasmic localization of CHMP7 and rescued the aberrant splicing of STMN2 (Al-Azzam et al., 2024). This suggests a potential link between endosomal dysfunction and NPC integrity, further highlighting the complexity of NCT deficits in C9orf72-ALS.

TDP-43, a nuclear RNA-binding protein crucial for splicing and RNA stability, is mislocalized to the cytoplasm in over 97% of ALS cases, where it forms insoluble aggregates that deplete nuclear TDP-43 and drive cellular toxicity (Cristi et al., 2023). Notably, C-terminal fragments (CTFs) of TDP-43 co-aggregate with multiple Nups, contributing to NCT defects (Chou et al., 2018). In addition, the dipeptide repeat protein poly-GR has been implicated in exacerbating TDP-43 aggregation. Specifically, poly-GR promotes the aggregation of endogenous TDP-43 by disrupting the localization of Nup98 and transmembrane POM121, both of which partially colocalize with poly-GR aggregates (Cook et al., 2020). Furthermore, poly-GR–induced mislocalization of Nup62 leads to the formation of insoluble Nup62:TDP-43 inclusions, which are widely detected in *postmortem* central nervous system CNS tissues of patients with C9orf72-associated ALS and sALS (Gleixner et al., 2022).

NPC defects extend beyond C9orf72 and TDP-43 pathology. In SOD1G93A mice, aging-dependent cytoplasmic accumulation of Nup107, Nup50, and GP210 in motor neurons mirrors findings in sALS patient tissues, where elevated cytoplasmic Nups correlate with disease progression (Chew et al., 2019; Shang et al., 2017). Similarly, FUS-mutant motor neurons exhibit aberrant Nup62 and POM121 clustering, which can be reversible by wild-type FUS expression, underscoring FUS’s role in maintaining NPC architecture (Lin et al., 2021). Furthermore, Fus WT/H509D mice exhibited progressive motor deficits with age, as assessed by the accelerating rotarod and DigiGait system (Okada et al., 2024). These impairments were accompanied by the loss of motor neurons, disruption of the nuclear lamina and nucleoporins, and increased DNA damage in spinal cord motor neurons. RNA sequencing of FUS-H517D hiPSC-derived lower motor neurons (hiPSC-LMNs) further demonstrated a significant downregulation of genes associated with the nuclear lamina and nucleoporin complexes. Collectively, these findings suggest that disruption of the nuclear lamina and nucleocytoplasmic transport machinery plays a critical role in the pathogenesis of ALS as a common downstream event.

Human genetic studies implicate NPC dysfunction as a driver of ALS. GLE1 variants, critical for mRNA export, are linked to ALS in French-Canadian populations (Kaneb et al., 2015), while Nup50 polymorphisms increase sALS risk (Megat et al., 2023). Functional studies reveal that Nup50 knockdown exacerbates G_4_C_2_ repeat toxicity in *Drosophila* and induces neurodegeneration in ALS models, indicating that even partial Nup deficiencies disrupt homeostasis (Freibaum et al., 2015). Collectively, these findings argue for a paradigm shift: NPC components are not passive bystanders but active contributors to ALS pathogenesis across genetic and sporadic subtypes.

The emerging centrality of NPC dysfunction in ALS pathogenesis offers transformative insights yet underscores critical unresolved questions. While diverse genetic drivers—from C9orf72 hexanucleotide expansions to TDP-43, SOD1, and FUS mutations—converge on NCT defects, fundamental gaps persist in understanding the hierarchy of events. For instance, does NPC disruption initiate proteostasis collapse by mislocalizing RNA-binding proteins like TDP-43, or does protein aggregation precipitate secondary NCT failure? This temporal ambiguity is exacerbated by a two-way interaction between Nup dysfunction and pathology: poly-GR displaces Nups, promoting TDP-43 aggregation, while TDP-43 fragments co-aggregate with Nup62 and Nup98, creating a vicious cycle. Similarly, human genetic evidence implicating GLE1 and Nup50 in ALS risk raises questions about how partial Nup deficiencies interact with aging or environmental stressors to tip homeostasis toward disease. Key mechanistic mysteries also surround CHMP7—while its nuclear accumulation drives Nup degradation, it remains unclear whether CHMP7 dysregulation is a primary defect or a secondary response to NPC stress. Addressing these questions demands advanced models that recapitulate the spatiotemporal progression of NCT failure, particularly in sporadic ALS where upstream triggers are elusive.

NPC dysfunction presents a promising intervention point for ALS therapy. Three key approaches emerge: (1) CHMP7 inhibition to prevent NPC degradation, (2) nucleoporin stabilization to maintain transport competence, and (3) RanGAP1 restoration to preserve the RanGTP gradient. Critical unanswered questions remain regarding the therapeutic window - can NPC-directed interventions be effective after disease onset, and to what extent can surviving neurons compensate for NPC damage? While patient-derived models validate NPC pathology, bridging gaps between experimental systems remains essential for translation. Targeting these fundamental transport defects may offer a unified treatment strategy capable of addressing multiple ALS subtypes simultaneously.

### Nuclear pore complex and AD

AD, the most prevalent neurodegenerative dementia, is defined by two pathological hallmarks: extracellular amyloid-beta (Aβ) plaques and intraneuronal neurofibrillary tangles (NFTs) composed of hyperphosphorylated Tau (Knopman et al., 2021). These aggregates drive synaptic loss, neuronal death, and cognitive decline, yet the mechanisms linking molecular pathology to cellular dysfunction remain incompletely resolved. Emerging evidence positions NPC dysfunction as a critical mediator of AD progression, with Tau aggregation directly impairing NCT and nuclear integrity through interactions with Nups.

Histopathological studies first implicated NPC abnormalities in AD. Early electron microscopic examinations of AD-affected brains revealed neurons riddled with NFTs, alongside abnormal nuclear structures frequently associated with the nuclear lamina and NPCs (Metuzals et al., 1988). Furthermore, hippocampal neurons from AD patients exhibit cytoplasmic accumulation of NTF2—a nuclear import factor—suggesting impaired NCT (Sheffield et al., 2006). Interestingly, importin α1, a key NCT mediator, selectively accumulates in Hirano bodies within AD hippocampal neurons but is absent from Aβ plaques, NFTs, and Lewy bodies (LBs) in PD, highlighting AD-specific NCT defects (Lee et al., 2006).

The interplay between Tau pathology and NPC dysfunction is now well-established (Diez et al., 2022). Hyperphosphorylated Tau directly interacts with central channel Nups, including Nup62 and Nup98, forming cytoplasmic co-aggregates that impair NCT (Eftekharzadeh et al., 2018). These aggregates sequester critical transport factors, exacerbating nuclear-cytoplasmic mislocalization of proteins and RNA. The reversibility of Nup98 mislocalization, achieved by reducing soluble Tau levels, underscores the therapeutic potential of targeting Tau to restore NCT and mitigate NFT formation (Eftekharzadeh et al., 2018). In HEK cells expressing the TauP^301L^ mutant, cytoplasmic mislocalization extends beyond central channel Nups to nuclear basket Nups (e.g., Nup153), transmembrane Nups (e.g., Pom121), and inner ring Nups (e.g., Nup155), indicating widespread NPC disassembly (Montalbano et al., 2020). These structural disruptions are accompanied by nuclear envelope invaginations invaded by microtubules, a phenomenon linked to mutant Tau-induced mechanical stress on the nuclear membrane (Paonessa et al., 2019). Such invaginations coincide with lamin A/C redistribution and heterochromatin loss, further implicating nuclear architecture collapse in AD pathogenesis (Montalbano et al., 2020). Collectively, these findings position NPC dysfunction as both a consequence and amplifier of Tau-driven toxicity, contributing to transcriptional dysregulation and genomic instability in AD.

Moreover, Aβ oligomers, a hallmark of Alzheimer’s disease, have been shown to disrupt the function of the NPC. Studies in AD mouse models reveal that intracellular Aβ accumulation correlates with a significant loss of key nucleoporins (Nup98 and Nup107), thereby compromising the nuclear permeability barrier and permitting aberrant bidirectional protein exchange that disrupts nuclear homeostasis (Bansal et al., 2025). This disruption not only impairs the regulated transport of proteins necessary for gene expression and stress responses but also sensitizes neurons to inflammatory mediators, contributing to necroptotic cell death (Bansal et al., 2025). These data suggests that both Aβ and tau contribute to AD pathology possibly through convergent mechanisms of NPC dysfunction.

NPC dysfunction in AD may arise through two interrelated mechanisms: (1) NCT failure in AD disrupts the nuclear import of key neuronal survival factors such as TDP-43, CREB-binding protein, and DNA repair enzymes, which are crucial for synaptic plasticity and genomic stability. This failure contributes to widespread transcriptional dysregulation and increased neuronal vulnerability; and (2) mislocalization of Nups, particularly Nup98 and Nup62, exacerbates proteostasis deficits by impairing autophagic and ubiquitin-proteasome clearance mechanisms, leading to the accumulation of toxic proteins such as Aβ and hyperphosphorylated Tau. Notably, NPC defects in AD are potentially reversible—restoring Nup98 localization has been shown to reduce soluble Tau levels, while stabilizing microtubules can mitigate nuclear envelope invagination, a pathological feature linked to nuclear instability in AD (Eftekharzadeh et al., 2018). These findings position NPC integrity as a promising therapeutic target in AD, with potential interventions including Nup-stabilizing agents to restore nuclear-cytoplasmic balance and small-molecule inhibitors designed to disrupt pathogenic Tau-Nup interactions, thereby slowing disease progression.

### Nuclear pore complex and HD

HD is an autosomal dominant neurodegenerative disorder caused by an expanded CAG trinucleotide repeat in the huntingtin (*HTT*) gene, leading to a toxic polyglutamine (polyQ) tract in the N-terminal region of the huntingtin protein (HTT) (McColgan and Tabrizi, 2018). This mutation triggers the formation of misfolded HTT aggregates, which accumulate as intranuclear inclusions, predominantly in striatal medium spiny neurons and cortical regions (Grima et al., 2017; Lange et al., 2021). These aggregates disrupt proteostasis, transcriptional regulation, and intracellular transport, culminating in progressive motor dysfunction (e.g., chorea), cognitive decline, and psychiatric disturbances (McColgan and Tabrizi, 2018). While neuronal loss in the striatum and cortex is a hallmark of HD, emerging evidence highlights NPC dysfunction as a pivotal contributor to pathogenesis, offering novel mechanistic insights into selective neuronal vulnerability.

Recent proteomic and mechanistic studies have illuminated the interplay between mutant HTT (mHTT) and NPC components. Notably, mHTT aggregates sequester critical Nups, including Nup62, Nup153, Nup214, and Nup358, impairing NPC structural integrity and function (Suhr et al., 2001). Strikingly, mHTT directly interacts with RanGAP1 and RAE1, an mRNA export factor, disrupting RanGTP gradients and mRNA trafficking (Grima et al., 2017). These perturbations are conserved across HD models, including R6/2 and zQ175 mice, and *postmortem* human brains (Brettschneider et al., 2015; Cornett et al., 2005). Additional studies reveal that mHTT’s toxicity extends beyond protein aggregates: mutant *HTT*mRNA forms nuclear foci that sequester RNA-binding proteins, while phosphorylated mHTT exhibits enhanced nuclear retention due to weakened binding to TPR (Cornett et al., 2005; Havel et al., 2011). Such nuclear accumulation of mHTT protein and RNA may synergistically disrupt mRNA surveillance and export, trapping transcripts essential for synaptic function and survival.

Intriguingly, *HTT* itself contains a proline-tyrosine nuclear localization signal (PY-NLS) recognized by karyopherins (Kapβ1/2), suggesting its role as a chaperone for nuclear transport receptors (NTRs) (Desmond et al., 2012). mHTT’s aberrant interactions with NTRs may compromise their ability to ferry cargoes—such as survival factors or stress-response proteins, thereby amplifying neuronal dysfunction (Langfelder et al., 2016; Shirasaki et al., 2012). This dual role of HTT in both NPC engagement and NTR regulation underscores its multifaceted contribution to HD pathology.

Despite these advances, the direct causal link between NPC defects and neurotoxicity remains unresolved. A critical question is whether NPC defects are primary drivers of HD or secondary consequences of proteostatic stress. The interplay between mHTT’s nuclear RNA foci, phosphorylated isoforms with enhanced nuclear retention, and Nup sequestration remains unclear. Do these processes synergize to disrupt mRNA surveillance, or do they represent parallel pathways converging on NPC failure? Addressing this requires temporally controlled studies in preclinical models to dissect the sequence of events linking mHTT accumulation to NPC breakdown.

### Nuclear pore complex and PD

PD is characterized neuropathologically by the progressive degeneration of dopaminergic neurons in the substantia nigra pars compacta, a region critical for motor control, accompanied by the accumulation of cytoplasmic inclusions, LBs (Chen X. et al., 2020; Wu et al., 2023). LBs are composed of misfolded proteins, including α-synuclein fibrils, ubiquitinated proteasomal components, and nuclear proteins such as RNA-binding proteins (RBPs) like TDP-43 and hnRNPA2/B1, as well as nuclear transport factors like Importin 7 (Xia et al., 2008). The aberrant sequestration of nuclear proteins within these aggregates implies a breakdown in NCT. While most PD cases are sporadic, 10%–15% are linked to genetic mutations, including those in PARK2 (encoding Parkin), an E3 ubiquitin ligase critical for protein quality control. Intriguingly, Parkin directly regulates the stability of Nup358/RanBP2, a cytoplasmic NPC component essential for nuclear import and export (Um et al., 2006). Wild-type Parkin promotes proteasomal degradation of Nup358, whereas pathogenic Parkin mutants fail to do so, leading to Nup358 accumulation and potentially impairing NPC function (Um et al., 2006). These findings suggest that loss-of-function Parkin mutations in PD may destabilize NPC architecture, disrupting NCT and contributing to neuronal vulnerability (Riaz et al., 2024).

Emerging evidence further implicates NCT dysregulation in PD through pathological mislocalization of α-synuclein, a presynaptic protein that aggregates in LBs. While α-synuclein is predominantly cytoplasmic, post-translational modifications—such as phosphorylation at Ser129 (Schell et al., 2009) or C-terminal truncation (Zhou et al., 2013)—enhance its nuclear translocation. Recent studies in transgenic mice expressing nuclear-targeted α-synuclein (NLS-α-synuclein) revealed striking PD-like phenotypes, including age-dependent motor deficits, dopaminergic neuron loss, and dysregulation of nuclear proteins involved in synaptic function (Geertsma et al., 2022). Once in the nucleus, α-synuclein interacts with histones and DNA repair proteins (Alagna et al., 2023; Chen V. et al., 2020), potentially interfering with chromatin remodeling and transcriptional regulation. Notably, α-synuclein aggregates sequester nuclear RBPs like TDP-43(Alagna et al., 2023; Chen V. et al., 2020), suggesting a feedforward loop where NCT impairment exacerbates proteostatic stress. However, one emerging question is whether α-synuclein aggregates directly obstruct NPC channels or disrupt NCT indirectly by depleting essential transport receptors. Addressing this will be crucial for determining whether α-synuclein-induced nuclear transport defects can be reversed pharmacologically. Additionally, the role of genetic factors such as Parkin mutations in NPC destabilization warrants further exploration. If Parkin loss-of-function mutations lead to persistent Nup358 accumulation and NPC dysfunction, targeting this pathway could offer novel therapeutic strategies.

### Nuclear pore complex and neurodegeneration: emerging hypotheses

NPC is essential for NCT and genome integrity, making its dysfunction a potential early driver of neurodegeneration. Given the post-mitotic nature of neurons, NPC components are prone to cumulative damage, leading to impaired transport of key transcription factors, toxic protein accumulation, and dysregulated stress responses. Recent evidence suggests that these disruptions are not merely consequences but early pathological events contributing to neurodegenerative diseases. Here, we propose multiple hypotheses to enlighten the area.

Disruption of NCT: the NPC is essential for the regulated trafficking of proteins and RNAs between the nucleus and cytoplasm. When the NPC is compromised, import and export processes become inefficient. Critical transcription factors and RNA-binding proteins may become mislocalized—for example, protective factors that normally reside in the nucleus might accumulate in the cytoplasm, while stress-related signals that need to be exported may be trapped in the nucleus. This mislocalization disrupts gene regulation and could initiate a cascade of dysfunction, ultimately impairing cell survival.

Accumulation of toxic protein aggregates: in many neurodegenerative diseases, proteins such as TDP43, mutant huntingtin, or tau aggregate abnormally. These aggregates can physically interfere with the architecture of the NPC or alter the dynamic behavior of FG-repeat nucleoporins that form the transport barrier. The resulting structural changes may further impair nucleocytoplasmic trafficking and exacerbate the accumulation of misfolded proteins, thereby creating a vicious cycle where transport defects promote protein aggregation and aggregated proteins further compromise NPC function.

Age-Related deterioration of NPC integrity: neurons are long-lived cells with limited capacity for NPC turnover. Over time, oxidative damage and other age-associated insults can degrade the more dynamic components (like FG-Nups) of the NPC, leading to a gradual loss of pore integrity. Such deterioration predisposes aged neurons to additional stress—when a neuron with an already “leaky” or deficient NPC faces further insults (e.g., inflammation or toxic protein buildup), the combined effects can tip the balance toward dysfunction and cell death.

Perturbed Signaling and Stress Response Pathways: beyond its role in transport, the NPC also participates in organizing chromatin and regulating gene expression. Disruptions in NPC structure may lead to misregulated signaling pathways—for instance, altered localization or activation of kinases like ERK or transcription factors such as CREB can impair the cell’s ability to mount appropriate stress responses. Such alterations may prevent the induction of protective genes and leave neurons more vulnerable to environmental and intracellular stressors.

## Lamin and neurodegenerative diseases

### Fundamental characteristics of lamin proteins

INM interacts with the nuclear lamina, a crucial meshwork of intermediate filaments that maintains the nuclear architecture. In mammalian cells, the nuclear lamina comprises four principal lamin proteins: two A-type lamin proteins (lamin A and C, splice variants of the *LMNA* gene) and two B-type lamin proteins (lamin B1 and B2, encoded by distinct genes *LMNB1* and *LMNB2*) (Burke and Stewart, 2013; Hachiya et al., 2021). The expression of A-type lamins is regulated by developmental stages, whereas B-type lamins are ubiquitously expressed in all cells. It is important to note that not all cells express the full complement of lamin proteins, yet each cell type exhibits a unique configuration and organization of these proteins. It has been known that lamin proteins play a critical role in DNA replication, transcription, and reparation, as well as in the control of the cell cycle (de Las Heras et al., 2013; Wong and Stewart, 2020). During somatic cell division, lamin A/C undergoes phosphorylation, resulting in the disassembly of its network structure. As the division process towards completion, lamin A/C is dephosphorylated, facilitating the rebuilding of a network structure (de Las Heras et al., 2013; Wong and Stewart, 2020). Concomitantly, LADs (lamina-associated domains), typically transcriptionally silent, along with other nuclear molecules, are subjected to rearrangement and redistribution within the cell (Alagna et al., 2023). LADs are regions of the genome that interact with the nuclear lamina. These domains are typically enriched in heterochromatin modifications such as histone H3 lysine nine methylation (H3K9me2/3), which contribute to a repressive chromatin environment. This dynamic remodeling of the nuclear architecture is essential for the proper restoration of chromatin structure and function following mitosis (Otsuka et al., 2023).

The mechanical properties of the nucleus, including its stiffness and flexibility, are largely determined by the composition of lamin proteins within the cell (Swift et al., 2013). However, the precise roles of nuclear lamins in nucleocytoskeletal connectivity and cellular mechanics remain elusive. Recent research conducted by Goldman’s group, utilizing a combination of advanced quantitative microscopy, micromechanical assays, and molecular biology techniques on mouse embryonic fibroblasts (MEFs), has provided new insights into these critical functions (Vahabikashi et al., 2022). Their findings revealed that A-type and B-type lamins interact differently with LINC complexes. A-type lamins engage with both filamentous actin and vimentin intermediate filaments through LINC complexes to modulate cortical and cytoplasmic stiffness as well as cellular contractility. Conversely, B-type lamins interact solely with vimentin intermediate filaments through LINC complexes to regulate cytoplasmic stiffness and contractility. Understanding these mechanisms provides valuable insights into how cells manage their mechanical properties in response to various physiological and environmental stimuli.

### Lamin B and neurodegenerative diseases

B-type lamins play a pivotal role in embryonic development, especially in the central nervous system (CNS), as evidenced by research in murine and human models (Evangelisti et al., 2022). Disruptions in lamin B homeostasis have been linked to severe outcomes, including lethality at birth, as well as abnormal cerebral cortex and cerebellar development (Coffinier et al., 2010; Coffinier et al., 2011). Homozygous deletion of both *LMNB1* and *LMNB2* genes in mice results in phenotypes similar to lissencephaly, characterized by simplified cortical folding and cerebellar hypoplasia (Coffinier et al., 2010; Vergnes et al., 2004). Overexpression of lamin B1 is also detrimental to mouse CNS development by impairing neural activity and myelination (Heng et al., 2013; Rolyan et al., 2015). These findings underscore the importance of lamin B homeostasis for proper brain development and suggest that maintaining appropriate levels of lamin B is essential for neuronal health.

Lamin B is abundantly expressed in neurons and critically implicated in AD pathogenesis (Frost, 2016). Mechanistically, tau pathology induces aberrant stabilization of actin filaments, impairing dynamic actin-nucleoskeleton interactions essential for nuclear stiffness (Frost, 2016). This disruption compromises the mechanical coupling between the nucleoskeleton and cytoskeleton, leading to nuclear envelope invagination, reduced lamin B levels, and persistent DNA damage in post-mitotic neurons (Koufi et al., 2023). These findings position AD within the emerging framework of laminopathies, disorders traditionally linked to nuclear envelope dysfunction, and highlight lamin B as a guardian of neuronal nuclear integrity. Targeting tau-induced lamin B dysregulation may offer novel therapeutic avenues to restore nuclear-cytoskeletal crosstalk and mitigate neurodegeneration.

Beyond AD, dysregulated Lamin B1 also contributed to the pathophysiology of HD (Alcalá-Vida et al., 2021). In HD patient hippocampi, lamin B1 depletion disrupts lamin-associated chromatin domains (LADs), regions crucial for heterochromatin organization (Alcalá-Vida et al., 2021). This loss increases chromatin accessibility, derepressing repetitive genomic elements and dysregulating transcription of neuronal survival genes. Intriguingly, restoring lamin B1 levels in the R6/1 mouse model of HD effectively rescue nuclear homeostasis as well as mitigates motor and cognitive impairments (Alcalá-Vida et al., 2021). These findings underscore the significance of lamin B1 as a pathogenic factor in HD and suggest its potential as a therapeutic target.

Alterations in lamin B expression have also been identified in PD with evidence spanning diverse models. In *Drosophila melanogaster*, neuronal lamin B reduction recapitulates PD hallmarks, including motor decline and dopaminergic synapse loss at Dorsal Longitudinal Muscles (DLM), linking nuclear fragility to circuit dysfunction (Oyston et al., 2018). Human studies reveal selective lamin B depletion in PD astrocytes, rendering them susceptible to senescence-associated secretory phenotypes (SASP), which propagate neuroinflammation and α-synuclein aggregation (Chinta et al., 2018). Intriguingly, PD patient-derived iPSCs harboring the LRRK2 (G2019S) mutation exhibit progressive lamin B1/B2 loss in neural stem cells (NSCs), accompanied by nuclear envelope folding defects (Liu et al., 2012). Hyperactive LRRK2 kinase phosphorylates lamin B, destabilizing the nuclear lamina and impairing NSC self-renewal—a defect reversible with LRRK2 inhibitors. These findings implicate lamin B as a convergence point for genetic and age-related stressors in PD, with therapeutic potential in modulating LRRK2 activity or lamin stability to preserve nuclear integrity across neuronal and glial populations.

In addition to its role in neurodegenerative diseases, Lamin B has been closely linked to cellular senescence, a state of irreversible cell cycle arrest associated with aging and age-related diseases. During senescence, Lamin B1 levels decrease, leading to significant changes in nuclear architecture, including nuclear envelope deformation, chromatin reorganization, and the formation of senescence-associated heterochromatin foci (SAHF) (Dreesen et al., 2013; Freund et al., 2012). Additionally, reduced Lamin B1 levels have been implicated in the activation of SASP, which promotes chronic inflammation and tissue dysfunction during aging. Given its critical role in nuclear integrity and genome organization, Lamin B1 downregulation serves as both a marker and a driver of cellular senescence, further underscoring its relevance in aging-related processes beyond neurodegeneration.

### Lamin A and neurodegenerative diseases

**FIGURE 4:**
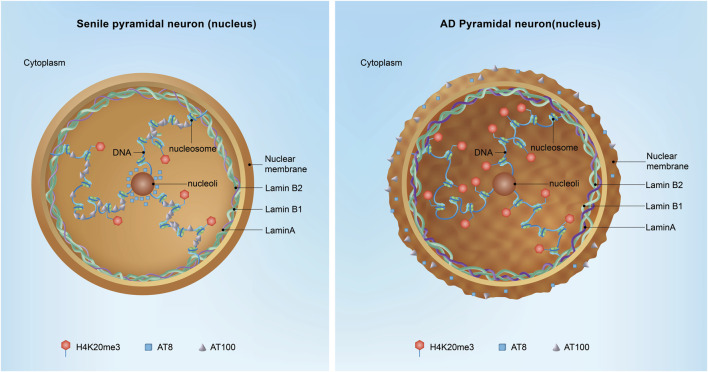
Nuclear Tau and neuronal AD transformation. In the aged hippocampus, nuclear Tau is particularly important for stabilizing the peripheral and nucleolar heterochromatin blocks. Phosphorylated Tau at specific epitopes, such as the AT100 and AT8 sites, acts as a molecular brake on the cell cycle, ensuring that vulnerable neurons remain quiescent and do not enter a state of division, which could lead to further DNA damage and cell death. However, in the context of Alzheimer’s disease (AD), the situation is markedly different. As Tau transitions from the nucleus to the cytoplasm, it loses its protective function, leading to the mislocalization and aggregation of Tau in the extracellular matrix, where it forms toxic protein aggregates. Additionally, Lamin A expression is increased associated with the augment of H4K20me3 in AD, suggesting the occurrence of dysregulation of the nuclear architecture and gene expression.

Unlike Lamin B, lamin A is expressed at minimal levels in the majority of neural and neuroendocrine cells. This distinct expression pattern from lamin B may explain why individuals with Hutchinson-Gilford Progeria Syndrome (HGPS), resulting from mutations in lamin A, do not exhibit the typical hallmarks of neurodegeneration (Gonzalo et al., 2017). Recent studies have revealed that in aged AD brains, post-mitotic hippocampal cells begin to express lamin A in the early course of the disease, leading to a more intricate nuclear lamin structure that includes lamin B1, B2, and A, compared to healthy aged neurons (Figure 4) (Gil et al., 2020; Mendez-Lopez et al., 2019). The incorporation of lamin A into the nucleus has been known to enhance the structural integrity of nuclei, conferring increased rigidity and viscosity; however, the underlying mechanisms responsible for the augmented expression of lamin A in AD remain elusive. These observations imply that a compromised nuclear lamin network plays a pivotal role in AD, with the upregulation of lamin A expression emerging as a distinguishing feature between healthy senescent and AD brains.

### Nuclear invagination

**FIGURE 5:**
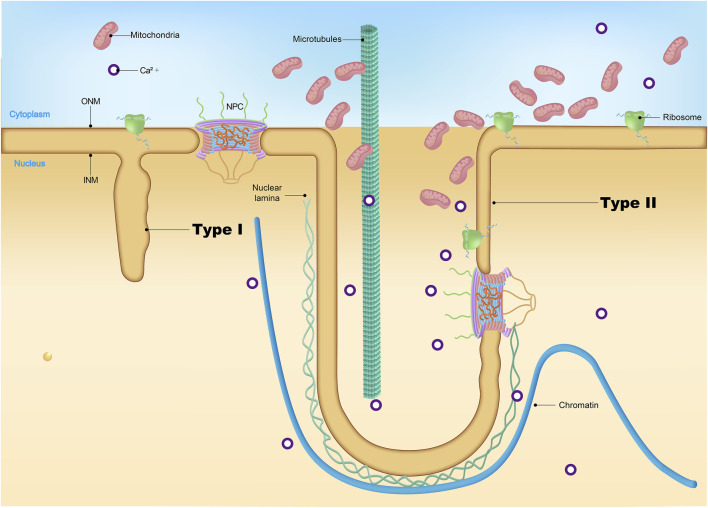
Nuclear invagination. While type I NR brings the lumen to deep nuclear locations it does not provide the cytoplasmic conduit characteristic of the type II NR channels. The complex topology of the type II channels, with a cytoplasmic interior that may be invaded by folds of outer nuclear membrane and often contains vesicles, cytoskeletal elements, higher concentrations of calcium and, in some cell types, mitochondria.

Nuclear invaginations are categorized into two types based on their structural characteristics (Malhas et al., 2011; Malhas and Vaux, 2014). Type II invaginations involve both the inner and outer nuclear membranes, are lined with nuclear pores, and encase a cytoplasmic core (Figure 5). These invaginations are frequently associated with nucleoli, regions known for intense ribosomal RNA synthesis. In contrast, the less common type I invaginations involve only the inner nuclear membrane and were recently found to contain lipid droplets. The functional roles of type I and type II nuclear invaginations in cellular processes remain elusive.

Interestingly, there is evidence suggesting that the increase of nuclear invaginations may be linked to physiological aging. Studies have shown that nuclei from the frontal cortex and hippocampus of aged marmosets exhibit a notable rise in nuclear invaginations compared to their younger counterparts (Honavar and Lantos, 1987). Similarly, increased nuclear invaginations have been observed in the pyramidal neurons of the hamster motor cortex and human cortical neurons with aging (Buschmann and LaVelle, 1983; Spoerri et al., 1981). In AD, it was reported that about 60% of neuronal nuclei in AD brains show nuclear invaginations, representing a threefold increase relative to the age-matched control brains (Frost et al., 2016). Moreover, we found that depleting PD-related LRRK2 caused nuclear invaginations during the aging process (Chen X. et al., 2020). Similar results are observed in other PD-related mouse models (Chen et al., 2022). Thus, disruptions in nuclear shape may aggravate neuronal dysfunction and accelerate the aging process. Yet, the precise molecular pathways governing these phenomena are complex and require further investigation to elucidate.

### Lamin and neurodegeneration: emerging hypotheses

How lamin defects precipitate neurodegeneration remains unclear; however, several hypotheses have been proposed. One suggests that laminopathies may bring out either excessively rigid or fragile NE, leading to the disruption of NE stability and the damage of NCT. Alternatively, lamin dysregulation may impair the expression of neuronal functional and survival genes through interfering with the interaction between chromatin and transcription factors, thereby dysregulating gene expression, particularly the genes pivotal to cell survival, differentiation, and metabolic processes, essential for neuronal functionality. Furthermore, lamin defects could impair the repair pathway of DNA damage, leading to genomic instability and mutation accumulation—a cardinal feature of neurodegenerative disorders. Elucidating these mechanisms may unearth innovative therapeutic avenues for lamin-related neurodegenerative diseases.

Current therapeutic strategies for LMNA-linked laminopathies include pharmacological agents targeting downstream cellular pathways. Notably, mTOR inhibitors like everolimus and farnesyltransferase inhibitors such as lonafarnib (DuBose et al., 2018; Macicior et al., 2021) show potential to ameliorate cellular dysfunction by enhancing autophagy-mediated clearance of toxic proteins (e.g., progerin) and restoring lamin post-translational processing. Repurposed drugs, including statins and NSAIDs, may stabilize the fragile NE by modulating membrane fluidity (Lichtenberger et al., 2012; Redondo-Morata et al., 2016), while proteasome inhibitors like bortezomib (Sogbein et al., 2024) could reduce premature degradation of misfolded lamin mutants, allowing time for proper folding.

Emerging approaches aim to directly correct the genetic or molecular defects. Adeno-associated virus (AAV)-mediated delivery of functional LMNA or LMNB1 genes seeks to reconstitute lamin networks, whereas CRISPR-based exon skipping strategies could bypass pathogenic mutations. Complementary to these, small-molecule NCT modulators such as KPT-350, which inhibit excessive nuclear export (Haines et al., 2015), may rebalance disrupted transport pathways. When combined with NE-stabilizing agents, these interventions could synergistically preserve neuronal homeostasis. Despite these advances, no cure exists, underscoring the need for combinatorial therapies targeting both NE mechanics and NCT dynamics. Current research emphasizes a multi-pronged strategy integrating pharmacological, gene-editing, and protein-stabilizing modalities to address the complex pathophysiology of laminopathies.

## Conclusion and perspective

**FIGURE 6:**
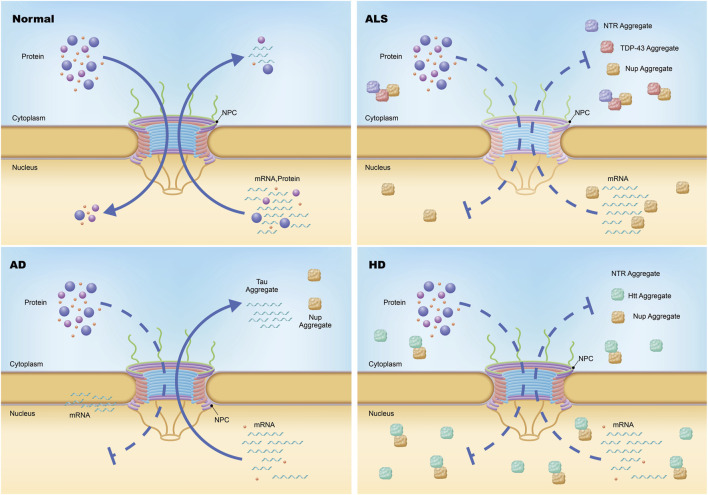
Nuclear pore complex pathology in neurodegeneration. Under physiological conditions, the transport of messenger RNA (mRNA) and proteins across the nuclear envelope is mediated by the nuclear pore complex (NPC), a highly selective gateway. In amyotrophic lateral sclerosis (ALS), specific nucleoporins (Nups) within the NPCs of neurons are diminished without altering the total count or structural integrity of the NPCs themselves, resulting in compromised import and export functions. Additionally, certain Nups and nucleocytoplasmic transport proteins are found to aggregate within the cytoplasm *via* an unidentified mechanism. In Huntington disease (HD), both the import and export processes mediated by the NPC are impeded, leading to defective import of proteins and export of mRNA. Moreover, a subset of Nups and nuclear pore-associated proteins involved in nucleocytoplasmic transport are sequestered in nuclear and cytoplasmic aggregates. In Alzheimer disease (AD), cytoplasmic and perinuclear accumulation of a small number of Nups occurs.

Dysfunction of the NE and NPCs has emerged as a hallmark of several age-related neurodegenerative diseases, including ALS, AD, HD, and PD (Figure 6; Table 1 and 2). As mislocalization and aggregation of proteins within the NPC gain recognition as critical pathological processes, it is essential to deepen our understanding of how these defects contribute to disease progression. However, several key questions remain unresolved, particularly regarding the precise functional consequences of NPC and NE abnormalities in different cell types and across diverse neurodegenerative conditions. This knowledge gap is especially pronounced in sporadic diseases, where the molecular pathways leading to NPC and NE dysfunction remain unclear.

**TABLE 1:** The alterations of Nup and NTR observed in neurodegenerative diseases (the phenotypes documented in at least two distinct sources are marked with bold font for emphasis).

Neurodegenerative disease	Protein affected	Alteration	Model system	References
Amyotrophic lateral sclerosis	Aladin	Mislocalization	Neuro-2A cells	PMID: 29311743
	Gle1	Cytoplasmatic mislocalization and aggregation	Neuro-2A cells	PMID: 29311743
	GP210	Reduced nuclear expression, nuclear aggregation, altered nuclear envelope distribution and increased cytoplasmic accumulation	Neuro-2A cells, transgenic mouse model, iPSNs and ALS patients	PMID: 29311743, 28344074, 32673563
	NDC1	Reduced nuclear expression	iPSNs	PMID: 32673563
	POM121	**Reduced nuclear expression**, reduced expression at the nuclear envelope, perinuclear aggregation and altered nuclear envelope distribution	Neuro-2A cells, mouse primary motor neurons, transgenic mouse model, human lymphoblast cells, iPSNs, and ALS patients	PMID: 29311743, 32878979, 34059832, 32673563, 34321318, 31444357
	RanGAP1	**Nuclear aggregation**, **altered nuclear envelope distribution** and increased cytoplasmic accumulation	*Drosophila* S2 cells, mouse primary cortical neurons, transgenic mouse model, mouse with AAV virus injection, iPSNs, and ALS patients	PMID: 26308891, 29311743, 28344074, 30767771
	RanGTPase	**Reduced nuclear/cytoplasmic ratio**	iPSNs	PMID: 26308891, 32673563, 34321318
	TPR	**Reduced nuclear expression** and cytoplasmic mislocalization	Transgenic *Drosophila* model, and iPSNs	PMID: 36130523, 32673563, 34321318
	XPO5	Mislocalization and aggregation	Neuro-2A cells	PMID: 29311743
	Nup35	Cytoplasmatic mislocalization and aggregation	Neuro-2A cells	PMID: 29311743
	Nup50	**Reduced nuclear expression**, nuclear aggregation and cytoplasmic mislocalization	transgenic *Drosophila* model, transgenic mouse model, iPSNs, and ALS patients	PMID: 28344074, 32673563, 34321318, 36130523
	Nup58	Cytoplasmatic mislocalization and aggregation	Neuro-2A cells	PMID: 29311743
	Nup62	Reduced nuclear expression, altered nuclear envelope distribution, cytoplasmatic mislocalization and **cytoplasmatic aggregation**	Neuro-2A cells, HEK293 cells, primary mouse motor neurons and human lymphoblast cells, iPSNs, and ALS patients	PMID: 29311743, 35697676, 34059832, 31444357
	Nup88	Cytoplasmatic mislocalization and cytoplasmatic aggregation	Neuro-2A cells,	PMID: 29311743
	Nup93	Reduced nuclear expression, cytoplasmatic mislocalization and **cytoplasmatic aggregation**	Neuro-2A cells, transgenic *Drosophila* model,	PMID: 29311743, 36130523
	Nup98	Reduced nuclear expression, perinuclear aggregation, and cytoplasmic mislocalization	Neuro-2A cells, transgenic *Drosophila* model, transgenic mouse model, iPSNs and ALS patients	PMID: 29311743, 32878979, 32673563, 36130523
	Nup107	Reduced nuclear expression, nuclear aggregation, **perinuclear aggregation**, and altered nuclear envelope distribution	Neuro-2A cells, transgenic *Drosophila* model, transgenic mouse model, iPSNs and ALS patients	PMID: 26308891, 29311743, 28344074, 32673563, 26308899
	Nup133	**Reduced nuclear expression**	iPSNs, and ALS patients	PMID: 32673563, 34321318
	Nup153	Reduced nuclear expression, and altered distribution at the nuclear envelope	Neuro-2A cells, primary mouse motor neurons, human lymphoblast cells, and iPSNs	PMID: 29311743, 34321318, 31444357
	Nup155	Cytoplasmatic mislocalization and aggregation	Neuro-2A cells	PMID: 29311743
	Nup160	Cytoplasmatic mislocalization and aggregation	Neuro-2A cells	PMID: 29311743
	Nup205	Nuclear aggregation, **perinuclear aggregation**, altered nuclear envelope distribution, cytoplasmatic mislocalization and **cytoplasmatic aggregation**	Neuro-2A cells, transgenic mouse model, iPSNs and ALS patients	PMID: 26308891, 28344074, 29311743
	Nup214	**Reduced expression**, altered distribution at the nuclear envelope, cytoplasmatic mislocalization and cytoplasmatic aggregation	Neuro-2A cells, transgenic *Drosophila* model, primary mouse motor neurons and human lymphoblast cells	PMID: 29311743, 36130523, 31444357
	Nup358	Reduced expression, altered distribution at the nuclear envelope, cytoplasmatic mislocalization and aggregation	Neuro-2A cells, primary mouse motor neurons and human lymphoblast cells	PMID: 29311743, 31444357
Alzheimer’s dementia	importin α1	Accumulated in Hirano bodies	AD patients	PMID: 17070506
	NTF2	Increased cytoplasmatic accumulation	AD patients	PMID: 16410748
	Nup50	Increased cytoplasmatic level	HEK cells	PMID: 32855391
	Nup62	Perinuclear co-aggregation with phospho-Tau	AD patients	PMID: 30189209
	Nup85	Increased cytoplasmatic level	HEK cells	PMID: 32855391
	Nup88	Increased cytoplasmatic level	HEK cells	PMID: 32855391
	Nup98	Perinuclear co-aggregation with phospho-Tau	Transgenic mouse model and AD patients	PMID: 30189209
	Nup107	Increased cytoplasmatic level	HEK cells	PMID: 32855391
	Nup133	Increased cytoplasmatic level	HEK cells	PMID: 32855391
	Nup153	Increased cytoplasmatic level	HEK cells	PMID: 32855391
	Nup155	Increased cytoplasmatic level	HEK cells	PMID: 32855391
	Nup214	Increased cytoplasmatic level	HEK cells	PMID: 32855391
Huntington disease	Gle1	Abnormal nuclear distribution and co-aggregation with mHTT	Transgenic mouse model	PMID: 28384474
	POM121	Co-aggregation with mHTT	HEK293	PMID: 11309410
	RanGAP1	**Co-aggregation with mHTT,** abnormal nuclear distribution, intranuclear accumulation, cytoplasmic mislocalization	Transgenic mouse model, iPSNs, and HD patients	PMID: 28384479, 28384474
	RanGTPase	Reduced nuclear/cytoplasmic ratio	Primary mouse cortical neurons and iPSNs	PMID: 28384479
	Nup62	**Co-aggregation with mHTT,** abnormal nuclear distribution	HEK293, transgenic mouse model, and HD patients	PMID: 11309410, 28384479
	Nup88	**Co-aggregation with mHTT,** abnormal nuclear distribution	HEK293, transgenic mouse model	PMID: 11309410, 28384479
	Nup153	Co-aggregation with mHTT	HEK293	PMID: 11309410
	Nup214	Co-aggregation with mHTT	HEK293	PMID: 11309410
	Nup358	Co-aggregation with mHTT	HEK293	PMID: 11309410

**TABLE 2:** Position and function of NPC components as described in the manuscript.

NPC component	Classification	Position	Function
Nup62	Structural Nup (Channel)	Central channel of the NPC	Forms part of the FG-repeat meshwork, regulates selective permeability of the NPC, and interacts with transport receptors to facilitate molecular movement across the pore.
Nup107	Structural Nup (Scaffold)	Cytoplasmic side of the NPC, part of Nup107-160 complex	Contributes to NPC assembly and stability, important for the formation of the central channel and maintaining structural integrity of the NPC.
POM121	Structural Nup (Membrane)	Outer nuclear membrane	Transmembrane protein that helps integrate the NPC into the nuclear envelope, contributing to NPC stability and facilitating nuclear transport.
Nup133	Structural Nup (Scaffold)	Part of Nup107-160 complex	Plays a role in NPC assembly and organization of the central channel.
Nup205	Structural Nup (Scaffold)	Near the central channel, part of Nup107-160 complex	Participates in the structural organization of the NPC, maintaining the pore’s integrity.
GP210	Structural Nup (Membrane)	Inner nuclear membrane	Stabilizes the NPC by interacting with the inner nuclear membrane and linking it to the NPC; may also play a role in NPC assembly and positioning.
Nup214	Structural Nup (Scaffold)	Cytoplasmic side of the NPC, part of the Nup214-Nup88 complex	Contributes to the NPC’s structural integrity, assists in nuclear import and export, and interacts with transport receptors and cargo proteins.
Nup358 (also known as RanBP2)	Structural Nup (Scaffold)	Cytoplasmic side of the NPC, forms part of the cytoplasmic fibrils	Functions in the regulation of Ran GTPase cycle and in nuclear transport by interacting with import/export receptors. Also plays a role in mitosis and NPC stability.
NDC1	Structural Nup (Membrane)	Inner nuclear membrane	Involved in NPC integration into the nuclear envelope and stabilizing the complex by interacting with other membrane-bound Nups.
GLE1	Functional Nup	Nuclear basket of the NPC	Plays a role in mRNA export, interacting with RNA helicase Dbp5 to drive RNA export through the NPC
RanGAP1	Functional Nup	Cytoplasm, near the NPC	Activates GTP hydrolysis on Ran, regulating the directionality of nucleocytoplasmic transport and disassembly of cargo-receptor complexes after nuclear import.
TPR	Functional Nup	Nuclear basket of the NPC	Involved in the final steps of nucleocytoplasmic transport, particularly in the disassembly of import/export complexes and regulating mRNA export.
Nup50	Structural/Functional Nup	Cytoplasmic basket of the NPC	Involved in disassembling importin-cargo complexes after nuclear import and interacting with FG-repeat Nups to facilitate transport receptor binding.
Nup153	Structural/Functional Nup	Nuclear basket of the NPC	Involved in nuclear transport, interacts with transport receptors, and plays a role in chromatin organization and gene regulation.

### How does NPC dysfunction lead to disease-specific pathophysiology?

To elucidate how NPC dysfunction contributes to disease-specific pathophysiology, a multi-modal approach integrating molecular profiling and dynamic functional analysis can be employed. By leveraging transcriptomic (RNA-seq) and proteomic techniques on *postmortem* brain tissues from individuals with ALS, HD, AD, and/or PD. researchers can systematically map disease-specific alterations in NPC components, such as NUPs and transport factors. This comparative analysis may reveal distinct molecular signatures, providing mechanistic insights into how NPC composition diverges across neurodegenerative conditions. To complement these static profiles, live-cell imaging in iPSC neurons or animal models enables real-time visualization of NCT dynamics. By tagging cargo proteins like RanGTP—a critical regulator of transport—or disease-associated proteins such as TDP-43 and Tau, researchers can track compartmentalization errors, such as nuclear accumulation of pathological aggregates or impaired export of RNA-binding proteins. Together, these strategies bridge molecular snapshots of NPC structure with functional transport deficits, uncovering how divergent NPC disruptions in the distinct neurodegenerative diseases drive unique pathological cascades, from protein mislocalization to neuronal dysfunction. This integrated framework advances the understanding of NPC biology as a nexus of disease-specific vulnerability.

### How do specific NPC defects define disease phenotypes?

The molecular mechanisms by which defects in NPC function contribute to distinct disease phenotypes are increasingly linked to disease-specific disruptions in NCT and neuronal vulnerability. In ALS, cytoplasmic mislocalization of TDP-43 coincides with impaired RNA export, while HD is characterized by polyglutamine-expanded huntingtin proteins that physically destabilize NPC architecture. AD, in contrast, features tau-driven nuclear membrane irregularities that compromise compartmental integrity. Emerging tools like proximity labeling (BioID or APEX2) enable spatial mapping of NPC-associated protein networks in these disease models, revealing how aberrant interactions may drive pathology. Notably, the selective susceptibility of specific neuronal populations—striatal spiny projection neurons in HD, motor neurons in ALS, and cortical neurons in AD—appears tied to cell-type-specific NPC dysregulation. Cutting-edge approaches combining single-nucleus ATAC-seq and RNA-seq are now unraveling how epigenetic and transcriptional landscapes unique to these neurons interact with NPC dysfunction, potentially explaining their heightened vulnerability. Together, these findings highlight how NPC defects manifest in disease-specific molecular cascades, shaped by both intrinsic protein interactions and the chromatin-environmental context of affected cells.

### Why are neurons more vulnerable?

Neurons exhibit a heightened vulnerability compared to other cell types, a phenomenon driven by the interplay of their unique NPC composition, activity-dependent stresses, and post-mitotic constraints. Unlike non-neuronal cells, neurons possess a distinct NPC proteome marked by selective expression of nucleoporins. These molecular signatures, identifiable through single-cell RNA sequencing, may confer structural or functional specializations that render neuronal NPCs inherently susceptible to stress. Compounding this vulnerability, neurons face dynamic physiological demands: synaptic activity and calcium signaling generate mechanical and metabolic strain that could destabilize NPC integrity over time. Advanced methodologies, such as coupling electrophysiological recordings with super-resolution microscopy, enable real-time observation of how heightened neuronal activity impacts NPC stability. Additionally, neurons lack the regenerative capacity of mitotic cells, which periodically renew NPCs during division. This post-mitotic limitation necessitates extraordinary durability of NPC components, a trait that can be quantified using pulse-chase labeling techniques like SNAP-tag or Halo-tag systems to track protein turnover rates. Collectively, the confluence of specialized NPC architecture, activity-induced wear, and an absence of mitotic renewal creates a unique susceptibility profile in neurons, predisposing them to NPC dysfunction and related degenerative pathways.

### Targeting NPC: emerging therapies for neurodegeneration

Therapeutic strategies targeting NPC dysfunction in neurodegenerative diseases such as ALS, HD, and AD are increasingly focused on preserving NPC integrity and nuclear transport efficiency. One promising approach involves the use of adeno-associated virus (AAV) vectors to overexpress protective NUPs in animal models, aiming to restore NPC structure and function, which could ameliorate pathological hallmarks like protein aggregation and neuronal degeneration. Complementing this, interventions to enhance NUP turnover—such as autophagy enhancers or immunotherapies—are being explored to bolster the clearance of cytotoxic aggregates, thereby maintaining NPC functionality under cellular stress. Oxidative damage, a key contributor to NPC instability, is being addressed through antioxidant therapies designed to shield redox-sensitive FG-Nups from harm, potentially slowing the deterioration of nuclear-cytoplasmic transport. Concurrently, molecular chaperones like HSP70 are under investigation for their ability to stabilize existing NPCs by preventing the misfolding of critical components, offering a dual mechanism of protection and structural support. These multifaceted strategies, spanning gene therapy, protein homeostasis, oxidative stress mitigation, and chaperone induction, collectively aim to delay disease progression by targeting overlapping pathways implicated in NPC dysfunction. By integrating these approaches, researchers hope to not only preserve neuronal health but also address the systemic cellular stressors that underlie neurodegenerative pathology, paving the way for combinatory therapies with synergistic benefits.
